# Sulodexide Protects Renal Tubular Epithelial Cells from Oxidative Stress-Induced Injury via Upregulating Klotho Expression at an Early Stage of Diabetic Kidney Disease

**DOI:** 10.1155/2017/4989847

**Published:** 2017-08-27

**Authors:** Yu Ning Liu, Jingwei Zhou, Tingting Li, Jing Wu, Shu Hua Xie, Hua-feng Liu, Zhangsuo Liu, Tae Sun Park, Yaoxian Wang, Wei Jing Liu

**Affiliations:** ^1^Key Laboratory of Chinese Internal Medicine of Ministry of Education and Beijing and Renal Research Institution of Beijing University of Chinese Medicine, Dongzhimen Hospital Affiliated to Beijing University of Chinese Medicine, Beijing 100700, China; ^2^Institute of Nephrology, Zhanjiang Key Laboratory of Prevention and Management of Chronic Kidney Disease, Guangdong Medical University, Zhanjiang, Guangdong 524001, China; ^3^The First Affiliated Hospital of Zhengzhou University, Zhengzhou, China; ^4^Chonbuk National University, Jeonju, Republic of Korea

## Abstract

The hypoalbuminuric effect of sulodexide (SDX) on diabetic kidney disease (DKD) was suggested by some clinical trials but was denied by the Collaborative Study Group. In this study, the diabetic rats were treated with SDX either from week 0 to 24 or from week 13 to 24. We found that 24-week treatment significantly decreased the urinary protein and HAVCR1 excretion, inhibited the interstitial expansion, and downregulated the renal cell apoptosis and interstitial fibrosis. Renoprotection was also associated with a reduction in renocortical/urinary oxidative activity and the normalization of renal klotho expression. However, all of these actions were not observed when SDX was administered only at the late stage of diabetic nephropathy (from week 13 to 24). In vitro, advanced glycation end products (AGEs) dose-dependently enhanced the oxidative activity but lowered the klotho expression in cultured proximal tubule epithelial cells (PTECs). Also, H_2_O_2_ could downregulate the expression of klotho in a dose-dependent manner. However, overexpression of klotho reduced the HAVCR1 production and the cellular apoptosis level induced by AGEs or H_2_O_2_. Our study suggests that SDX may prevent the progression of DKD at the early stage by upregulating renal klotho expression, which inhibits the tubulointerstitial injury induced by oxidative stress.

## 1. Introduction

Diabetic kidney disease (DKD), a common and serious microvascular complication of diabetes mellitus (DM), is the leading cause of end-stage renal disease (ESRD) and renal failure as well as the major determinant of morbidity and mortality in DM patients. Although the pathogenesis of DKD is to some extent well known, including the activation of the rennin-angiotensin system (RAS), formation of advanced glycation end products (AGEs), and activation of protein kinase C (PKC), agents targeting these pathways may not completely prevent the progression of DKD. Therefore, it is an urgent need to establish new therapeutic options and targets to inhibit the progression of DKD [1].

Recently, rather than an orthodox eye to glomerular damage, accumulated studies provide new insight into tubular epithelial cell (TEC) injury in DKD, since renal tubule also plays a key role even in the pathogenesis of DKD [2]. During this process, oxidative stress is a crucial mediator to activate the DKD-associated signaling pathway [3]. Also, DKD is an aging-related disease characterized by accelerated renal intrinsic cell senescence, in which the aging that occurred in a tubule is more typical [4]. Klotho, expressed mainly in renal tubules, is one of the most important antiaging genes. And a decreased tubular klotho expression is found at the early stage of DKD in patients and rodents [5, 6]. Notably, the renoprotective role of klotho has been implicated in various acute and chronic kidney diseases including DKD [7–9], indicating the functional importance of antiaging in DKD. Furthermore, oxidative stress is one of the most important etiologies involved in cellular senescence, especially in the state of DM [6]. Therefore, the new target against klotho and oxidative stress is promisingly to provide a reference for DKD.

Sulodexide (SXD), a highly purified glycosaminoglycan that offers antithrombotic and profibrinolytic activity, is shown to prevent cells against various injuries. A large number of studies have strongly suggested the antiproteinuric and renoprotective action of sulodexide in type 1 and 2 diabetes patients [10, 11]. However, recent micro- and macroalbuminuria (Sun-MICRO and MACRO) studies dispute these results based on the multicentered trials in which SDX does not show obvious benefit on DKD [12]. Therefore, to clarify which patients could benefit most from SDX administration and what factors impact on SDX's efficacy, further studies are recommended [10]. Interestingly, it is reported that SXD offers antioxidant and antisenescent actions in cultured endothelial cells [13]. We have also reported that, via reducing oxidative activity, SDX exhibits protective effects on the peripheral nerve in diabetic complications [14]. It is possible through antioxidative stress and antiaging effects, SDX protects TECs from DKD-induced injury. This hypothesis was tested in experimental animals and cultured primary TECs in this study.

## 2. Material and Methods

### 2.1. Animals and Experimental Design

Male Sprague-Dawley rats weighing 220 to 240 g each were housed in a 12 h light-/dark-altered room at a constant temperature of 24°C, with food and water available ad libitum. Diabetes was induced by a single intraperitoneal injection of streptozotocin (60 mg/kg body weight; Sigma, St. Louis, MO, USA) dissolved in 0.1 mol/L citrate buffer (pH 4.5). The control rats were injected with an equal volume of citrate buffer. One week after the verification of diabetes, diabetic and nondiabetic rats were stochastically divided into 3 and 2 groups (*n* = 5–7 per group), respectively. Sulodexide (supplied by Asia Pharm., Korea) was dissolved in an appropriate volume of water and administered orally at 10 mg/kg/day to one diabetic group (DKD + 24SDX group) and one nondiabetic group (SDX group) in the nighttime for 24 weeks [14]. Another diabetic group was administered SDX (10 mg/kg/day) from week 13 to week 24 (DKD + 12SDX group). Diabetic (DKD group) and nondiabetic (CON group) control rats received the equal volume of vehicle within the same time. Water was offered in the daytime, and food was available ad libitum during the entire experimental period to all 5 groups. Body weight, food intake, and tail blood glucose were measured every 2 weeks after 8 h of fasting throughout the study period. Hemoglobin A1c (HbA1c) was determined by an aminophenyl-boronate-agarose affinity chromatographic method (Glyc-Affin GHb; Seikagaku Kogyo, Tokyo, Japan) in weeks 12 and 24. Experimental procedures were approved by the Ethics Committee of Beijing University of TCM and performed in accordance with the National Academies Guiding Principles for the Care and Use of Laboratory Animals, 8th edition.

### 2.2. Urinary and Plasmatic Parameters

After overnight fasting of the rats in weeks 12 and 24, blood samples were collected at 8 h postmeal via the tail vein and plasma was prepared. And the urine from each rat was collected with a metabolic cage (Nalgene; Sybron, Bend, OR, USA) as described previously [15]. Urine albumin concentration was measured by a time-resolved fluorometric immunoassay (Feng Hua Bioengineering Corporation, Guangzhou, China). Urinary 8-hydroxy-deoxyguanosine (8-OhdG) and HAVCR1 concentrations were measured by enzyme-linked immunosorbent assay (ELISA) kits purchased from Japan Institute for the Control of Aging (Shizuoka, Japan) and Cosmo Bio (Tokyo, Japan), respectively. Plasma insulin level was measured using an ELISA kit (Linco Research, St. Charles, Missouri, USA). Plasma and urine creatinine and urea concentrations were assayed with an automatic biochemistry analyzer (Olympus, Tokyo, Japan). Creatinine clearance was calculated as an index of glomerular filtration rate (GFR).

### 2.3. Kidney Cytoplasmic Lysate and Homogenate Analysis

In week 24, all the animals were killed, renal cortices were rinsed and weighed, and the cytoplasmic fractions were prepared as previously described [16]. TGF-*β*1 was quantified using the Quantikine Rat TGF-*β*1 immunoassay kit (R&D Systems, Minneapolis, MN, USA) according to the manufacturer's instructions.

### 2.4. Cell Culture and Treatments

Primary renal proximal tubule epithelial cells were obtained from ScienCell (Carlsbad, CA, USA) and were grown as suggested by the manufacturer. To mimic the diabetic kidney disease, the cells were exposed to 0, 25, 50, and 50 *μ*g/mL nonglycated control bovine serum albumin (Co-BSA) or AGE-BSA (BioVision, Mountain View, CA, USA) for 24 h. To mimic the pathological process of oxidative stress-induced kidney disease clinically, the cells were exposed to 0, 0.3, 0.3, and 0.4 mM H_2_O_2_ for 24 h. To study the action of klotho *in vitro*, the primary tubule epithelial cells were infected with an adenovirus encoding the klotho gene virus [17] (3.2 × 10^7^ pfu) (provided by GeneChem, Shanghai, China) for 24 h before exposure to AGE-BSA or H_2_O_2_, and then, the cell samples and culture supernatant were collected for the following experiment.

### 2.5. Determination of Renal Oxidative and Antioxidative Stress Biomarkers

The activity of SOD (U/gram tissue) and GPX (nmol/min/gram tissue) was determined by an ELISA reader (Absorbance Microplate Reader ELx 800 TM BioTek®, USA) using the commercially available kits (Cayman Chemical Company, Ann Arbor, MI, USA). MDA (Cayman Chemical Company) and total ROS (eBioscience, San Diego, CA, USA) were also measured according to the manufacturer's instructions.

### 2.6. Histological Analysis

For histological study, kidneys were fixed with 4% paraformaldehyde and embedded in JB-4. 1.5 *μ*m thick sections were stained by Masson's trichrome. Briefly, the fraction of the renal cortex occupied by interstitial tissue (INT%) was quantitatively evaluated in Masson-stained sections using a point-counting technique under a 176-point grid [15, 18]. Photomicrographs were captured using a Carl Zeiss Axioskop2 plus microscope (Carl Zeiss, Göttingen, Germany) and a digital camera (AxioCam HRC, Carl Zeiss, Göttingen, Germany) with the final magnification at 200x. From each tissue, 30 tubular fields (5 sections) were counted in a blinded fashion by two independent investigators.

### 2.7. Western Blot Assay

Kidney tissues were homogenized with RIPA buffer and protease inhibitors. 50 micrograms of total protein was loaded in a stacking polyacrylamide gel and resolved on a polyacrylamide gel with biotinylated molecular weight standard markers. The samples were then transferred to a 0.2-micron nitrocellulose membrane. After blocking for 1 h, the blots were incubated overnight at 4°C with rabbit antibodies raised against cleaved-caspase-3 (Cell Signaling Technology, Beverly, MA, USA), rabbit anti-klotho antibody (Abcam), and mouse anti-*α*-SMA (Abcam). After washing, the membranes were probed with secondary goat anti-mouse IgG-HRP or goat anti-rabbit IgG-HRP-linked antibody (Santa Cruz Biotechnology, Santa Cruz, CA, USA) for 1 hour at room temperature. The bands were detected using an enhanced chemiluminescence (ECL) solution (Amersham Biosciences, Uppsala, Sweden) and followed by exposure to X-ray film. The optical density for quantification was determined using Bandscan 4.0 software (Glyko, USA).

### 2.8. Real-Time PCR

Total RNA was extracted from isolated renocortical tissues using TRIzol reagent (Invitrogen, Carlsbad, CA, USA) and treated with RNase-free DNase (Invitrogen, Carlsbad, CA, USA). First-strand complementary DNA (cDNA) was generated with random primers by reverse transcriptase (TaKaRa, Otsu, Japan). The PCR reaction was carried out using a SYBR Green master mix kit and the ABI Prism 7900HT Sequence Detection System (Applied Biosystems, Foster City, CA, USA). All reactions were conducted in triplicate as described previously [17]. The obtained value was adjusted with a control gene (*β*-actin) and expressed as a percentage of the value in normal control extracts. The sequences of the primers were as follows (forward and reverse, resp.): 5′-CGT GAA TGA GGC TCT GAA AGC-3′ and 5′-GAG CGG TCA CTA AGC GAA TAC G-3′ (klotho) and 5′CGTGAAAAGATGACCCAGATCA-3′ and 5′-TGGTACGACCAGAGGCATACAG-3′ (*β*-actin).

### 2.9. Statistical Analysis

Data are presented as the mean ± standard error of the mean (SEM), and a one-way analysis of variance with Duncan's post hoc test was used. Data were considered statistically significant if *p* < 0.05. Statistical analysis was performed using SPSS 12.0 software.

## 3. Results

### 3.1. SDX Reduced the Urinary Albumin and Protein Excretion in Diabetic Rats

Food intake, fasting blood glucose, and HbA1c levels were significantly higher, whereas body weight and plasma insulin levels were markedly lower in diabetic rats compared with nondiabetic animals. SDX treatment did not significantly attenuate the changes (Table 1). Diabetic rats exhibited increased urinary albumin and protein excretion, high serum creatinine, and blood urea nitrogen levels; enhanced albumin/creatinine and kidney/body weight ratios; and decreased creatinine clearance at week(s) 12 and/or 24. 24-week treatment with SDX, but not 12-week treatment (from week 13 to 24), significantly reduced the albuminuria, proteinuria, and albumin/creatinine ratio at both time points (Table 2).

### 3.2. SDX Offered Antioxidative and Antiaging Effects on the Kidney of Diabetic Rats

**Figure 1:**
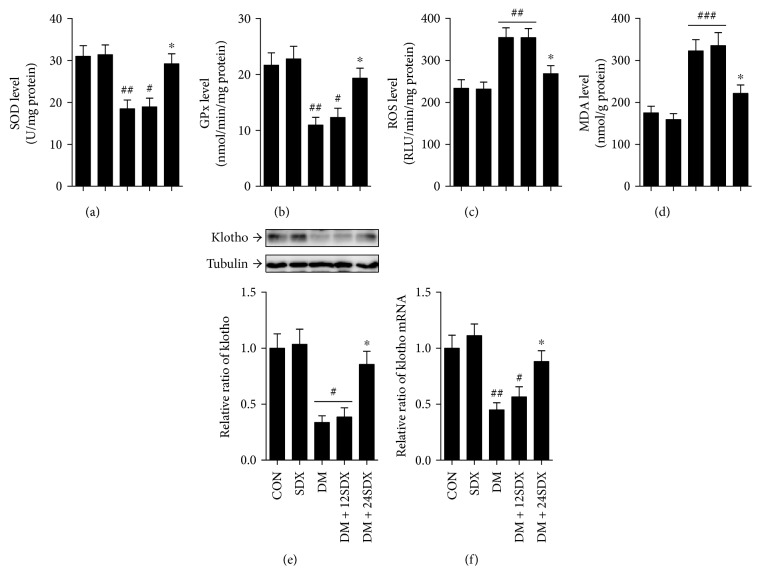
Effects of SDX on oxidative stress biomarker and klotho expression in the kidney of nondiabetic and diabetic rats. (a–d) Bar graphs show the changes of SOD (a), GPx (b), ROS (c), and MDA (d) levels in the renal cortex of each group. (e, f) Protein and mRNA levels of klotho are analyzed by Western blot and real-time PCR in the rat renal cortex. Data are expressed as fold of control. CON = vehicle-treated nondiabetic group; SDX = treated with 10 mg/kg/day sulodexide. ^#^*p* < 0.05, ^##^*p* < 0.01, and ^###^*p* < 0.001 versus CON. ^∗^*p* < 0.05 versus vehicle-treated diabetic control (DM).

Firstly, we found that the SOD and GPx levels were significantly lower in the renal cortex of the untreated diabetic group than in the renal cortex of the normal control group. However, treatment with SDX for 24 weeks significantly increased the antioxidant levels in diabetic rats (Figures 1(a) and 1(b)). Accordingly, the ROS and MDA levels were found to increase in the renal cortex of diabetic rodents. The levels of oxidative stress markers were notably reduced by 24-week SDX administration (Figures 1(c) and 1(d)). Also, we found that DM induced a marked decrease in renocortical protein and mRNA expression of klotho in DM rats. Either protein or mRNA level of klotho was augmented by 24-week treatment with SDX (Figures 1(e) and 1(f)). However, these actions were not found when treated from week 13 to 24.

### 3.3. SDX Attenuated Cellular Apoptosis and Interstitial Fibrosis in the Kidney of Diabetic Rats

**Figure 2:**
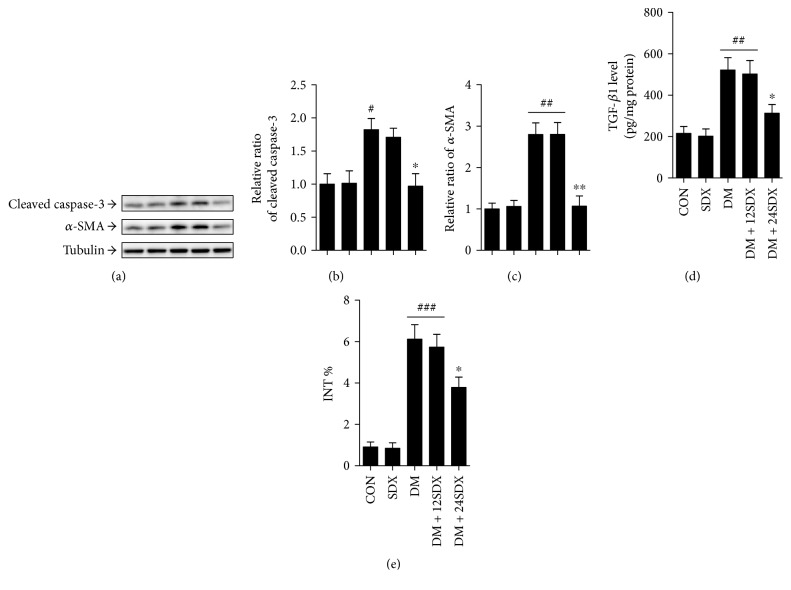
Effect of SDX on cellular apoptosis and interstitial fibrosis in the kidney of nondiabetic and diabetic rats. (a) The protein levels of cleaved caspase-3, *α*-SMA, and tubulin are analyzed by Western blot in the renal cortex of rats. (b, c) Densitometry is performed for quantification, and the ratio of cleaved caspase-3 or *α*-SMA to tubulin is expressed as fold of control. (d) TGF-*β*1 level is assayed in renal cortex lysates. (e) Bar graph shows the fraction of the renal cortex occupied by interstitial tissue (INT%). CON = vehicle-treated nondiabetic group; SDX = treated with 10 mg/kg/day sulodexide. ^#^*p* < 0.05, ^##^*p* < 0.01, and ^###^*p* < 0.001 versus CON. ^∗^*p* < 0.05 and ^∗∗^*p* < 0.01 versus vehicle-treated diabetic control (DM).

In our study, an elevated cleaved caspase-3 protein level, as determined by Western blot analysis, was noted in the renal cortex of the diabetic group indicating increased cellular apoptosis (Figures 2(a) and 2(b)). Interestingly, this protein level was significantly lowered after 24-week treatment with SDX. To evaluate the interstitial fibrosis, the expression of *α*-SMA and the fraction of the renal cortex occupied by interstitial tissue were measured by Western blot and Masson staining, respectively. As shown in Figures 2(a), 2(c), and 2(e), *α*-SMA protein level and INT% were higher in the DM group than in the control group, which could be markedly attenuated by SDX treatment for 24 weeks. Also, the similar pattern was obtained when assessing the renocortical TGF-*β*1 protein level, which had been reported to link epithelial-mesenchymal transition (EMT) and interstitial fibrosis (Figure 2(d)). And the renoprotection was also not observed when treated from week 13 to 24.

### 3.4. SDX Reduced Urinary 8-OHdG and HAVCR1 Excretions in Diabetic Rats

**Figure 3:**
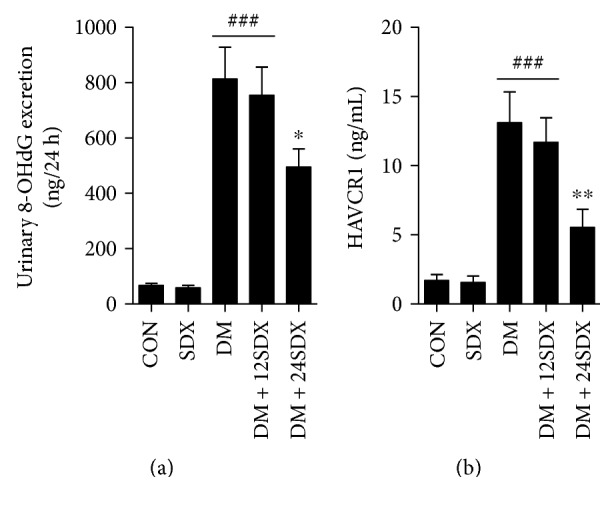
Effect of SDX on 8-OHdG and HAVCR1 excretions in nondiabetic and diabetic rats. (a, b) Bar graphs show the changes of 8-OHdG and HAVCR1 excretions in each group. Data are expressed as mean ± SEM. CON = vehicle-treated nondiabetic group; SDX = treated with 10 mg/kg/day sulodexide. ^###^*p* < 0.001 versus CON. ^∗^*p* < 0.05 and ^∗∗^*p* < 0.01 versus vehicle-treated diabetic control (DM).

Urinary 8-OHdG and HAVCR1 excretions were remarkably higher in diabetic rats than in controls (Figures 3(a) and 3(b)). However, the increased urinary 8-OHdG and HAVCR1 levels were significantly suppressed by 24-week SDX administration, but not by 12-week administration at the late stage of DKD.

### 3.5. AGE-BSA Triggered PTEC Senescence Partially via Upregulating Oxidative Stress

**Figure 4:**
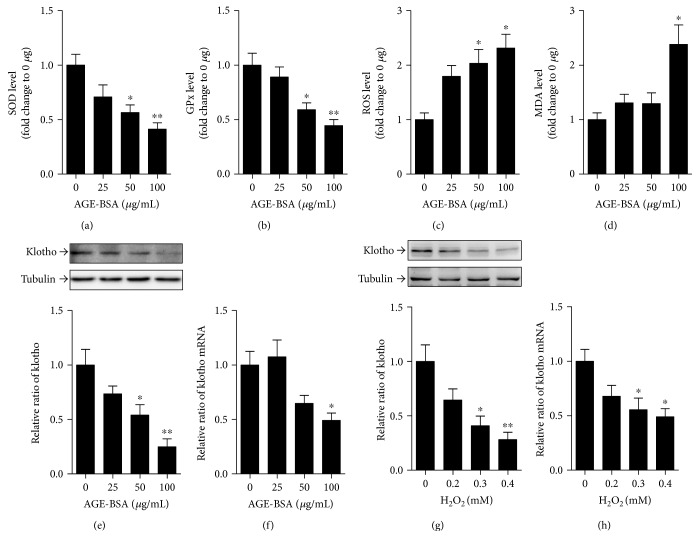
Effect of AGE-BSA on oxidative stress and klotho expression in cultured PTECs. (a–d) Bar graphs show the changes of SOD (a), GPx (b), ROS (c), and MDA (d) levels in PTECs after exposure to 0, 25, 50, or 100 *μ*g/mL AGE-BSA. (e, f) Protein and mRNA levels of klotho are measured by Western blot and real-time PCR in cultured PTECs after exposure to different concentrations of AGE-BSA. (g, h) Protein and mRNA levels of klotho are assessed by Western blot and real-time PCR in cultured PTECs after exposure to 0, 0.2, 0.3, or 0.4 mM H_2_O_2_. Data are expressed as fold of control. ^∗^*p* < 0.05 and ^∗∗^*p* < 0.01 versus 0 *μ*g/mL AGE-BSA.

Next, we studied the effects of different concentrations of AGE-BSA on oxidative stress and klotho expression. Exposure of PTECs to AGE-BSA dose-dependently decreased SOD and GPx levels but increased ROS level compared with the exposure to 0 *μ*g/mL AGE-BSA (Figures 4(a), 4(b), and 4(c)). And MDA level also rose after treatment with 100 *μ*g/mL AGE-BSA (Figure 4(d)). Furthermore, we found that exposure to AGE-BSA led to a decrease in the protein or mRNA expression of klotho, in a dose-dependent manner (Figures 4(e) and 4(f)). These alterations were not observed when exposed to the same concentration of nonglycated control BSA (Co-BSA) (data not shown). To explore the causal relationship between oxidative stress activation and cellular senescence, klotho expression was assessed after exposure of PTECs to hydrogen peroxide (H_2_O_2_). Interestingly, we found that exposure to 0.3 or 0.4 mM H_2_O_2_ markedly decreased the protein and mRNA levels of klotho (Figures 4(g) and 4(h)), suggesting that AGE-BSA-triggered cellular senescence was at least partially via activating oxidative stress.

### 3.6. Klotho Overexpression Protected PTECs from Injury Induced by AGE-BSA

**Figure 5:**
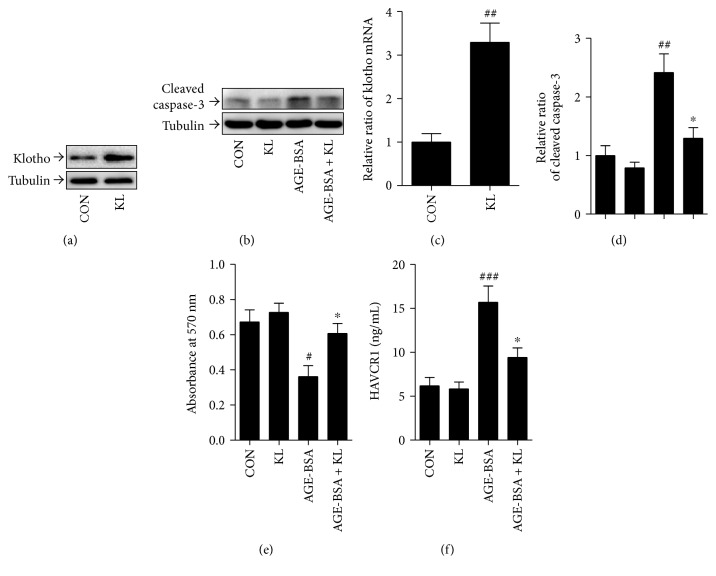
Effect of klotho overexpression on PTEC injury induced by AGE-BSA. (a, c) Protein and mRNA expression of klotho in PTECs after transfection with klotho plasmid (KL) or the control vector (CON). (b, d) The protein levels of cleaved caspase-3 and tubulin are analyzed by Western blot in PTECs exposed to 100 *μ*g/mL AGE-BSA or vehicle after transfection with either KL plasmid or empty vector control. Densitometry is performed for quantification, and the ratio of cleaved caspase-3 to tubulin is expressed as fold of control. (e) Cell viability was assessed by the MTT assay as described in (b). (f) Bar graphs show the HAVCR1 excretions as described in (b). ^#^*p* < 0.05, ^##^*p* < 0.01, and ^###^*p* < 0.001 versus CON. ^∗^*p* < 0.05 versus AGE-BSA.

Next, we further demonstrated the critical role of klotho on PTEC injury after exposure to AGE-BSA by upregulating klotho expression. As expected, transfection with klotho plasmid successfully increased klotho expression as assessed by Western blot and real-time PCR (Figures 5(a) and 5(c)). Exposure to AGE-BSA significantly increased cellular apoptosis (*p* < 0.01, Figures 5(b) and 5(d)) and inhibited cell viability (*p* < 0.05, Figure 5(e)), characterized by an elevated cleaved caspase-3 protein level and reduced absorbance in the MTT assay, respectively. Also, AGE-BSA treatment enhanced the cellular HAVCR1 secretion (*p* < 0.001, Figure 5(f)). However, after klotho transfection, such effects were markedly attenuated in cultured PTECs.

### 3.7. Klotho Overexpression Protected PTECs from Injury Induced by H_2_O_2_

**Figure 6:**
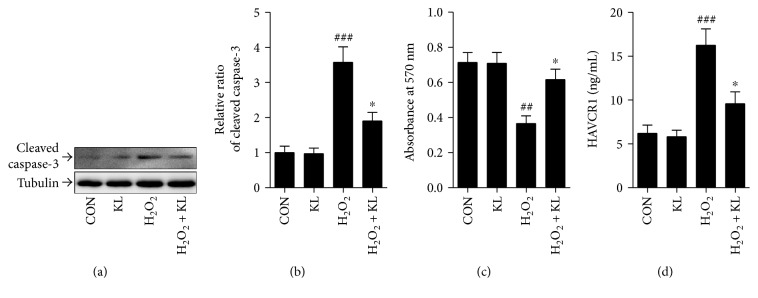
Effect of klotho overexpression on PTEC injury induced by H_2_O_2._ (a) The protein levels of cleaved caspase-3 and tubulin are analyzed by Western blot in PTECs after exposure to 0.3 mM H_2_O_2_. (b) Densitometry is performed for quantification, and the ratio of cleaved caspase-3 to tubulin is expressed as fold of control. (c, d) Bar graphs show the changes of cell proliferation, characterized by the MTT assay, and HAVCR1 excretions in PTECs after exposure to H_2_O_2_. CON = transfected with the control vector; KL = transfected with klotho plasmid. ^##^*p* < 0.01 and ^###^*p* < 0.001 versus CON. ^∗^*p* < 0.05 versus KL.

Finally, we studied the potential renoprotection of klotho during the injury induced by H_2_O_2_. PTEC apoptosis assessed by cleaved caspase-3 expression was elevated, but cellular proliferation characterized by the MTT assay was reduced after exposure to H_2_O_2_ compared with vehicle (Figures 6(a), 6(b), and 6(c), *p* < 0.001 and *p* < 0.01, resp.). However, these changes were alleviated in PTECs that overexpressed klotho (*p* < 0.05). Also, exposure to H_2_O_2_ resulted in an obvious enhancement of HAVCR1 excretion (Figure 6(d), *p* < 0.001). As expected, transfection of PTECs with klotho plasmid attenuated the HAVCR1 release (*p* < 0.05).

## 4. Discussion

Although current first-line treatment for DKD is proposed to inhibit the angiotensin-converting enzyme or block the angiotensin II receptor, only an approximate 2-year reprieve from ESRD is gained in type 2 diabetic patients with nephropathy [19, 20]. Since 1994, SDX and glycosaminoglycans (GAGs) have long believed to reduce urinary proteins and arrest DKD progression based on the clinical study of Solini et al. [21]. After that, with small patients and short duration, quite a few pilot studies indicate favourable results of SDX and GAGs on both micro- and macroalbuminuria in DKD [22]. In agreement with the previous reports, a multicenter international trial also proved the hypoalbuminuric effect of SDX, which was not even hindered by the usage of the angiotensin-converting enzyme inhibitor (ACEI), in the Diabetic Nephropathy and Albuminuria Sulodexide (DiNAS) Study published in 2002 [23]. However, in 2012, the Collaborative Study Group (CSG) demonstrated that sulodexide failed to offer renoprotection in DKD in 2 placebo-controlled double-blinded sulodexide trials with more patient numbers (micro- and macroalbuminuria, Sun-MICRO and MACRO) [24]. Could we sentence SDX for DKD to “another one bites the dust” as suggested by the editorial [12]? Is there any difference between Sun-MICRO/MACRO protocol and previous clinical studies, and what influences the difference? All of these await further investigation.

One of the striking differences is that the patients enrolled in the Sun-MICRO/MACRO study have severer nephropathy at baseline than those enrolled in previous reports, since the patients recruited in the GSG trial were most with chronic kidney disease (CKD) 3-4 even after the use of ACEI or ARBs [12, 25], while the subjects in DiNAS were mainly with CKD 2 [24]. It is also indicated that SDX's renoprotection only occurs at the early stage of CKD [26]. So, in this study, we differentiate SDX's action in the early stage of DKD from the late stage of DKD in experimental rat models. Interestingly, early-12-week treatment with SDX offered hypoalbuminuric, hypoproteinuric, and urea nitrogen-decreasing effects, but nonrenoprotection was observed when giving the agent in the onset phase of obvious proteinuria. Accompanying a decrease in urinary protein excretion, an alleviation of tubular cell apoptosis and the prevention of EMT and tubulointerstitial fibrosis were also noted post early-12-week SDX treatment, suggesting that SDX delays the disease progression at the early stage of DKD but not at the late stage of DKD. On the other hand, it also hints that TECs should be focused when exploring the action of SDX, not only with an eye on glomerular cells (e.g., endothelial cell). Then, what is the mechanism underlying SDX's protection for TEC? It is reported that SDX reduces senescence-related cellular injury [13]. Notably, the expression of the antiaging gene klotho is reduced in the onset of DKD, which exacerbates the early nephropathy [5, 27]. Also, the soluble klotho is decreased only in early-stage CKD since it would increase thereafter [28], indicating that klotho deficiency mainly results in the tubular alteration at the early stage of DKD. Indeed, we found that the expression of tubular klotho was decreased and upregulating klotho was one of the most important mechanisms underlying SDX's function only at the early stage of DKD. In addition, our data suggested that the oxidative stress had close relationship with and even accounted for the tubular downregulation of klotho *in vivo* and *in vitro*, respectively. However, the alteration of klotho expression was likely influenced mainly by oxidative stress in the onset of DKD since its causality was not observed at the late stage of DKD. More interestingly, in addition to attenuating the TEC injury induced by AGE-BSA, klotho overexpression increased the cellular resistance to H_2_O_2_ insult, indicating that klotho's renoprotection was partially through antioxidative action which agreed with previous studies [29].

In addition, the patients enrolled in the Sun-MICRO/MACRO study have already received maximal ACEI or ARBs [24], which differs from previous trials. Although a further hypoalbuminuric effect has been noted after adding SDX therapy to ACEI in a subgroup in DiNAS [23], as well as another GAG, enoxaparin cannot counter the renal hemodynamic changes including an increase in mean arterial pressure and a decrease in renal plasma flow post angiotensin II (AngII) infusion in DKD patients [30], suggesting the SDX activity is partially independent of the ACEI/ARB effects; whether SDX and ACEI/ARB have totally different pharmacological targets on hypoalbuminuria remains unclear. Actually, the klotho gene is first identified during the study on spontaneous hypertension, and klotho deficiency has close relationship with hypertension [31]. Importantly, ameliorating the klotho downregulation is one of the important mechanisms for ACEI/ARB to retard the progression of DKD [32, 33]. Furthermore, the antioxidative stress effects should be also considered when discussing the renoprotection of ACEI/ARB in patients with DKD [34]. All of these pharmacological targets of ACEI/ARB treatment were addressed by SDX therapy in our study, indicating that SDX and ACEI/ARB regulate, at least partially, some mutual pathways to treat DKD, which should be noted when demonstrating nonrenoprotection of SDX in the GSG study.

In conclusion, we note that SDX offers renoprotection at the early stage of DKD rather than at the late stage of DKD. The observed activity of SDX is probably attributable to upregulation of klotho expression, which could further increase the TEC response to oxidative stress in DKD. Therefore, the different populations of DKD patients and the addressed targets of pharmacological action should be considered when exploring the clinical interest of SDX.

## Figures and Tables

**Table 1:** Effects of sulodexide on body weight, food intake, blood glucose, HbA1c, and plasma insulin in nondiabetic and diabetic rats in week 24.

Group	CON	SDX	DKD	DKD + 12SDX	DKD + 24SDX
Body weight (g)	588 ± 30	575 ± 33	229 ± 14^###^	230 ± 14	225 ± 12
Food intake (g/day)	27.4 ± 1.8	26.9 ± 1.9	47.5 ± 3.2^###^	46.2 ± 3.5	46.9 ± 2.9
Blood glucose (mg/dL)	109 ± 4.85	102 ± 4.77	483 ± 3.98^###^	462 ± 6.14	479 ± 6.89
HbA1c (%)	4.41 ± 0.15	4.58 ± 0.19	12.89 ± 0.59^###^	11.56 ± 0.92	12.76 ± 0.85
Insulin (ng/dL)	1.83 ± 0.13	1.79 ± 0.15	0.29 ± 0.06^###^	0.35 ± 0.07	0.33 ± 0.06

Data are expressed as mean ± SEM. ^###^*p* < 0.001 as compared with CON. CON = nondiabetic control; DKD = diabetic kidney disease control; 12SDX = treated with 10 mg/kg/day sulodexide from week 13 to 24; 24SDX = treated with 10 mg/kg/day sulodexide from week 0 to 24.

**Table 2:** Effects of sulodexide on renal functions in nondiabetic and diabetic rats.

	Week	CON	SDX	DKD	DKD + 12SDX	DKD + 24SDX
Serum creatinine (mg/dL)	12	0.47 ± 0.05	0.44 ± 0.04	0.95 ± 0.11^##^	1.01 ± 0.10	0.82 ± 0.11
	24	0.55 ± 0.06	0.56 ± 0.05	1.35 ± 0.18^###^	1.09 ± 0.10	0.94 ± 0.10
Blood urea nitrogen (mg/dL)	12	27.1 ± 3.0	26.2 ± 3.6	54.3 ± 5.9^##^	55.2 ± 4.9	32.7 ± 4.2^∗^
	24	33.6 ± 3.4	31.3 ± 3.4	63.8 ± 6.7^##^	59.8 ± 5.9	44.4 ± 4.4
Urinary albumin (*μ*g)	12	245 ± 24	261 ± 26	954 ± 119^###^	938 ± 88	518 ± 64^∗∗^
	24	256 ± 22	245 ± 20	1344 ± 149^###^	1288 ± 134	788 ± 84^∗∗^
Urinary protein (mg)	12	12.5 ± 1.4	11.8 ± 1.3	21.9 ± 2.1^##^	20.5 ± 1.9	13.7 ± 1.4^∗^
	24	13.0 ± 1.7	13.4 ± 1.8	26.4 ± 2.3^##^	26.1 ± 2.7	16.0 ± 1.8^∗^
Albumin (*μ*g)/creatinine (mg)	12	15.3 ± 1.4	16.2 ± 1.5	64.7 ± 6.9^###^	65.7 ± 6.8	41.8 ± 5.0^∗^
	24	19.5 ± 3.0	19.4 ± 2.6	84.1 ± 8.6^###^	80.8 ± 7.8	52.6 ± 5.3^∗^
Creatinine clearance (mL/min)	12	1.65 ± 0.14	1.58 ± 0.14	0.99 ± 0.11^#^	0.91 ± 0.09	1.34 ± 0.14
	24	1.46 ± 0.11	1.49 ± 0.15	0.73 ± 0.09^##^	0.72 ± 0.11	1.21 ± 0.14
Kidney/body weight × 1000	24	4.86 ± 0.57	4.44 ± 0.58	8.58 ± 0.75^#^	8.82 ± 0.81	7.82 ± 0.74

Data are expressed as mean ± SEM. ^#^*p* < 0.05, ^##^*p* < 0.01, and ^###^*p* < 0.001 as compared with CON; ^∗^*p* < 0.05 and ^∗∗^*p* < 0.01 as compared with DKD. CON = nondiabetic control; DKD = diabetic kidney disease control; 12SDX = treated with 10 mg/kg/day sulodexide from week 13 to 24; 24SDX = treated with 10 mg/kg/day sulodexide from week 0 to 24.

## References

[1] Soetikno V., Arozal W., Louisa M. (2014). New insight into the molecular drug target of diabetic nephropathy. *International Journal of Endocrinology*.

[2] Tonolo G., Cherchi S. (2014). Tubulointerstitial disease in diabetic nephropathy. *International Journal of Nephrology and Renovascular*.

[3] Lee H. B., Yu M. R., Yang Y., Jiang Z., Ha H. (2003). Reactive oxygen species-regulated signaling pathways in diabetic nephropathy. *Journals of the American Society of Nephrology*.

[4] Verzola D., Gandolfo M. T., Gaetani G. (2008). Accelerated senescence in the kidneys of patients with type 2 diabetic nephropathy. *American Journal of Physiology-Renal Physiology*.

[5] Asai O., Nakatani K., Tanaka T. (2012). Decreased renal α-klotho expression in early diabetic nephropathy in humans and mice and its possible role in urinary calcium excretion. *Kidney International*.

[6] Cheng M. F., Chen L. J., Cheng J. T. (2010). Decrease of klotho in the kidney of streptozotocin-induced diabetic rats. *Journal of Biomedicine & Biotechnology*.

[7] Hu M. C., Kuro-o M., Moe O. W. (2010). Klotho and kidney disease. *Journal of Nephrology*.

[8] Zununi Vahed S., Nikasa P., Ardalan M. (2013). Klotho and renal fibrosis. *Nephro-urology Monthly*.

[9] Huang J. S., Chuang C. T., Liu M. H., Lin S. H., Guh J. Y., Chuang L. Y. (2014). Klotho attenuates high glucose-induced fibronectin and cell hypertrophy via the ERK1/2-p38 kinase signaling pathway in renal interstitial fibroblasts. *Molecular and Cellular Endocrinology*.

[10] Gaddi A. V., Cicero A. F., Gambaro G. (2010). Nephroprotective action of glycosaminoglycans: why the pharmacological properties of sulodexide might be reconsidered. *International Journal of Nephrology and Renovascular Disease*.

[11] Cicero A. F., Ertek S. (2010). Preclinical and clinical evidence of nephro- and cardiovascular protective effects of glycosaminoglycans. *Archives of Medical Science*.

[12] House A. A., Weir M. A. (2011). Sulodexide for diabetic nephropathy: another one bites the dust. *American Journal of Kidney Diseases*.

[13] Suminska-Jasinska K., Polubinska A., Ciszewicz M., Mikstacki A., Antoniewicz A., Breborowicz A. (2011). Sulodexide reduces senescence-related changes in human endothelial cells. *Medical Science Monitor*.

[14] Jin H. Y., Lee K. A., Song S. K. (2012). Sulodexide prevents peripheral nerve damage in streptozotocin induced diabetic rats. *European Journal of Pharmacology*.

[15] Liu W. J., Xie S. H., Liu Y. N. (2012). Dipeptidyl peptidase IV inhibitor attenuates kidney injury in streptozotocin-induced diabetic rats. *The Journal of Pharmacology and Experimental Therapeutics*.

[16] Kuhad A., Chopra K. (2009). Attenuation of diabetic nephropathy by tocotrienol: involvement of NFkB signaling pathway. *Life Sciences*.

[17] Kato Y., Arakawa E., Kinoshita S. (2000). Establishment of the anti-klotho monoclonal antibodies and detection of klotho protein in kidneys. *Biochemical and Biophysical Research Communications*.

[18] Jepsen F. L., Mortensen P. B. (1979). Interstitial fibrosis of the renal cortex in minimal change lesion and its correlation with renal function. A quantitative study. *Virchows Archiv A, Pathological Anatomy and Histology*.

[19] Lewis E. J., Hunsicker L. G., Clarke W. R. (2001). Renoprotective effect of the angiotensin-receptor antagonist irbesartan in patients with nephropathy due to type 2 diabetes. *The New England Journal of Medicine*.

[20] Brenner B. M., Cooper M. E., de Zeeuw D. (2001). Effects of losartan on renal and cardiovascular outcomes in patients with type 2 diabetes and nephropathy. *The New England Journal of Medicine*.

[21] Solini A., Carraro A., Barzon I., Crepaldi G. (1994). Therapy with glycosaminoglycans lower albumin excretion rate in non-insulin dependent diabetic patients. *Diabetes, Nutrition & Metabolism*.

[22] Abaterusso C., Gambaro G. (2006). The role of glycosaminoglycans and sulodexide in the treatment of diabetic nephropathy. *Treatments in Endocrinology*.

[23] Gambaro G., Kinalska I., Oksa A. (2002). Oral sulodexide reduces albuminuria in microalbuminuric and macroalbuminuric type 1 and type 2 diabetic patients: the Di.N.A.S. randomized trial. *Journal of the American Society of Nephrology*.

[24] Lewis E. J., Lewis J. B., Greene T. (2011). Sulodexide for kidney protection in type 2 diabetes patients with microalbuminuria: a randomized controlled trial. *American Journal of Kidney Diseases*.

[25] Packham D. K., Wolfe R., Reutens A. T. (2012). Sulodexide fails to demonstrate renoprotection in overt type 2 diabetic nephropathy. *Journal of the American Society of Nephrology*.

[26] Rossini M., Naito T., Yang H. (2010). Sulodexide ameliorates early but not late kidney disease in models of radiation nephropathy and diabetic nephropathy. *Nephrology, Dialysis, Transplantation*.

[27] Lin Y., Kuro-o M., Sun Z. (2013). Genetic deficiency of anti-aging gene klotho exacerbates early nephropathy in STZ-induced diabetes in male mice. *Endocrinology*.

[28] Kacso I. M., Bondor C. I., Kacso G. (2012). Soluble serum klotho in diabetic nephropathy: relationship to VEGF-A. *Clinical Biochemistry*.

[29] Banerjee S., Zhao Y., Sarkar P. S., Rosenblatt K. P., Tilton R. G., Choudhary S. (2013). Klotho ameliorates chemically induced endoplasmic reticulum (ER) stress signaling. *Cellular Physiology and Biochemistry*.

[30] Benck U., Haeckel S., Clorius J. H., van der Woude F. J. (2007). Proteinuria-lowering effect of heparin therapy in diabetic nephropathy without affecting the renin-angiotensin-aldosterone system. *Clinical Journal of the American Society of Nephrology*.

[31] Zhou X., Chen K., Lei H., Sun Z. (2015). Klotho gene deficiency causes salt-sensitive hypertension via monocyte chemotactic protein-1/CC chemokine receptor 2-mediated inflammation. *Journal of the American Society of Nephrology*.

[32] Zanchi C., Locatelli M., Benigni A. (2013). Renal expression of FGF23 in progressive renal disease of diabetes and the effect of ACE inhibitor. *PLoS One*.

[33] Karalliedde J., Maltese G., Hill B., Viberti G., Gnudi L. (2013). Effect of renin-angiotensin system blockade on soluble klotho in patients with type 2 diabetes, systolic hypertension, and albuminuria. *Clinical Journal of the American Society of Nephrology*.

[34] Nakamura A., Shikata K., Nakatou T. (2013). Combination therapy with an angiotensin-converting-enzyme inhibitor and an angiotensin II receptor antagonist ameliorates microinflammation and oxidative stress in patients with diabetic nephropathy. *Journal of Diabetes Investigation*.

